# Clove (*Syzygium aromaticum*) and its bioactive constituent eugenol alleviate psoriatic inflammation by modulating the IL-36/IL-17A axis: a proteomic and mechanistic study

**DOI:** 10.3389/fphar.2026.1839501

**Published:** 2026-05-26

**Authors:** Tin-Yun Ho, Hsin-Yi Lo, Chiao-Che Chen, Hui-Chi Huang, Chien-Yun Hsiang

**Affiliations:** 1 School of Chinese Medicine & Graduate Institute of Chinese Medicine, China Medical University, Taichung, Taiwan; 2 Department of Health and Nutrition Biotechnology, Asia University, Taichung, Taiwan; 3 Department of Microbiology and Immunology, China Medical University, Taichung, Taiwan

**Keywords:** eugenol, interleukin-17A, interleukin-36, proteomics, psoriasis, *Syzygium aromaticum*

## Abstract

**Introduction:**

Clove (*Syzygium aromaticum*) is traditionally used for inflammatory skin disorders; however, its specific molecular mechanisms in psoriasis remain poorly defined. This study aimed to evaluate the efficacy of clove extract and its major component, eugenol, and to identify their molecular targets.

**Methods:**

We used an imiquimod (IMQ)-induced mouse model to assess the effects of oral clove extract and eugenol. Proteomic alterations were identified via isobaric tags for relative and absolute quantitation-based liquid chromatography-tandem mass spectrometry and bioinformatics. The specific interaction with the interleukin-17A (IL-17A)/IL-17 receptor A (IL-17RA) interface was validated through competitive binding assays and molecular docking, with further confirmation via immunohistochemistry.

**Results:**

Eugenol was identified as the major constituent of clove extract (65.8%). Clove extract and eugenol significantly alleviated psoriasiform lesions, reducing scaling scores and epidermal thickness. Proteomic analysis revealed that both treatments reversed IMQ-induced inflammatory signatures by modulating the interleukin-36 (IL-36) and IL-17 signaling pathways, specifically increasing IL-36 receptor antagonist expression while suppressing IL-36 levels. *In vitro* assays demonstrated that both treatments inhibited IL-17A–IL-17RA binding in a dose-dependent manner, supported by molecular docking indicating potential interactions between eugenol and IL-17A residues. Immunohistochemical analysis further confirmed reduced nuclear factor-κB (NF-κB) activation, decreased IL-17A expression, and diminished CD11b^+^ granulocyte infiltration in psoriatic skin.

**Conclusion:**

Clove extract and its major bioactive constituent eugenol exert significant anti-psoriatic effects through coordinated modulation of the IL-36/IL-17A inflammatory axis and suppression of NF-κB signaling. These findings provide mechanistic insight into the traditional use of clove in inflammatory skin disorders and highlight eugenol as a key bioactive mediator with potential as a complementary therapeutic strategy for psoriasis.

## 1 Introduction

Psoriasis is a chronic inflammatory skin disorder characterized by erythematous, scaly plaques resulting from excessive keratinocyte proliferation and epidermal thickening (Griffiths et al., 2021). It affects approximately 0.09%–11.43% of the global population and imposes substantial physical, psychological, and economic burdens (Wang et al., 2024a). Its pathogenesis is primarily driven by the dysregulated interleukin-23 (IL-23)/T helper 17 (Th17) axis, where interleukin-17 (IL-17) promotes epidermal hyperplasia and inflammatory cell infiltration (Ghoreschi et al., 2021; Wu et al., 2024). Additionally, the IL-36 cytokine family has recently emerged as a critical amplifier of this psoriatic inflammation (Sachen et al., 2022).

While biologics targeting IL-17 (e.g., secukinumab, bimekizumab) or the interleukin-36 (IL-36) receptor (e.g., spesolimab) have demonstrated high clinical efficacy (Armstrong and Read, 2020; Gonçalves et al., 2025), their long-term application is often hindered by high costs, accessibility constraints, and potential loss of responsiveness (Ferrara et al., 2024; Lin et al., 2026; Wang and Li, 2026). Consequently, there is a sustained interest in identifying accessible, low-toxicity small molecules that can serve as alternative or adjunctive therapies (Dabholkar et al., 2021).

Clove, the dried flower bud of *Syzygium aromaticum* (L.) Merr. & L.M. Perry, is widely used in traditional medical systems for the treatment of inflammatory and infectious conditions. In traditional Chinese medicine, clove (Dingxiang) is recorded in the *Ben Cao Gang Mu* for conditions described as “wind-toxicity swellings” and suppurative lesions, which are consistent with inflammatory skin disorders. Similarly, in Ayurveda and other ethnomedical practices, clove and its essential oil have been used to treat skin infections, wounds, and inflammatory dermatoses due to their antiseptic and analgesic properties (Batiha et al., 2020; Gil et al., 2026; Tariq et al., 2025). These consistent ethnopharmacological applications suggest that clove may possess therapeutic potential for inflammatory skin diseases such as psoriasis.

Clove contains a high proportion of eugenol, a phenolic compound that accounts for the majority of its essential oil (Batiha et al., 2020) and is responsible for many of reported anti-inflammatory, antioxidant, and antimicrobial activities (Gil et al., 2026; Haro-Gonzalez et al., 2021). Despite these well-documented bioactivities, the therapeutic relevance of clove and eugenol in psoriasis remains unclear. In particular, it is unknown whether they modulate key pathogenic pathways such as the IL-36/IL-17 axis that drive psoriatic inflammation.

To address this gap, the present study investigated the anti-psoriatic potential of clove (standardized concentrated Dingxiang extract granules) and its major constituent eugenol using an imiquimod (IMQ)-induced psoriasis-like mouse model. Histopathological evaluation and isobaric tags for relative and absolute quantitation (iTRAQ)-based liquid chromatography-tandem mass spectrometry (LC-MS/MS) proteomic profiling were performed to identify key pathways involved. Furthermore, IL-17A–IL-17 receptor A (IL-17RA) binding assays, molecular docking, and immunohistochemistry (IHC) were employed to elucidate the molecular targets and signaling mechanisms underlying the anti-psoriatic effects of clove and eugenol.

## 2 Materials and methods

### 2.1 Reagents and chemicals

All chemicals, unless otherwise stated, were purchased from Sigma-Aldrich (St. Louis, MO, United States). Eugenol (purity 99%) was dissolved in absolute ethanol to prepare a 100 mM stock solution. Acetonitrile, trifluoroacetic acid, and ethanol were obtained from Merck (Darmstadt, Germany). IMQ cream (Aldara®) was purchased from 3M Pharmaceuticals (St. Paul, MN, United States). Petroleum jelly (Vaseline) and Fc-tagged IL-17RA protein were obtained from Acros Organics (Pittsburgh, PA, United States). Recombinant human IL-17A and IL-17RA proteins were purchased from Sino Biological (Wayne, PA, United States). Goat anti-human IgG Fc antibody conjugated with horseradish peroxidase (HRP) (AP113P) and mouse monoclonal antibody against total p65 (MAB3026) were purchased from Millipore (Temecula, CA, United States). Rabbit polyclonal antibody against IL-17A (bs-1183R) and rabbit monoclonal antibody against CD11b (ab133357) were obtained from Bioss (Woburn, MA, United States) and Abcam (Cambridge, United Kingdom), respectively. The detailed information of antibodies used in this study is listed in Supplementary Table S1.

### 2.2 Herbs and high-performance liquid chromatography (HPLC) analysis

Standardized concentrated Dingxiang extract granules, derived from the dried flower bud of *Syzygium aromaticum* Merr. & L.M. Perry, were purchased from a GMP-certified traditional Chinese medicine manufacturer (Sun Ten Pharmaceutical Co., Taipei, Taiwan). For experimental use, 1 g of clove granules was extracted with 10 mL of ethanol by continuous shaking at 25 °C for 24 h. After centrifugation at 12,000 xg for 10 min, the supernatant was collected and filtered to obtain the stock solution. Eugenol (1 mg/mL in ethanol) was freshly prepared before each experiment.

The phytochemical profile of clove extract was analyzed using a Shimadzu HPLC system equipped with a diode array detector (HPLC-DAD; Shimadzu, Kyoto, Japan), as described previously (Ho et al., 2023). Chromatographic separation was performed with a mobile phase of solvent A (0.1% trifluoroacetic acid in water) and solvent B (100% acetonitrile) at a flow rate of 1.0 mL/min. The gradient elution increased linearly from 0% to 60% solvent B over 30 min. The injection volume was 20 μL, and detection was performed at 280 nm.

### 2.3 Animal experiment

Female BALB/cByJ mice (5–6 weeks old, weighing 19–20 g) were purchased from the National Center for Biomodels (Taipei, Taiwan) and housed in the China Medical University Animal Center under standard conditions (12 h light/dark cycle; controlled temperature and humidity) with free access to food and water. All procedures were approved by the Institutional Animal Care and Use Committee of China Medical University (Permit No. CMUIACUC-2022-130) and conducted in accordance with the U.S. National Institutes of Health Guidelines for the Care and Use of Laboratory Animals (NIH Publication No. 85–23, revised 1996).

A total of 30 mice were randomly assigned to six groups (n = 5 per group): (1) mock (Vaseline); (2) IMQ (IMQ only); (3) 50 mg/kg clove (IMQ +50 mg/kg clove); (4) 100 mg/kg clove (IMQ +100 mg/kg clove); (5) 200 mg/kg clove (IMQ +200 mg/kg clove); (6) 100 mg/kg eugenol (IMQ +100 mg/kg eugenol). Psoriasiform inflammation was induced by daily topical application of 62.5 mg Vaseline or IMQ cream to the shaved dorsal skin (2 × 2 cm^2^) for seven consecutive days (Ho et al., 2022). Clove (50, 100, or 200 mg/kg) or eugenol (100 mg/kg) was administered orally once daily for seven consecutive days concurrently with IMQ. The severity of desquamation (scaling) on the back skin was blindly scored on a scale of 0–4 (0 = none, 4 = severe). On day 8, mice were anesthetized with 4% isoflurane (Baxter, Deerfield, IL, United States) and euthanized. Skin samples were collected for histopathological staining and protein extraction.

### 2.4 Histopathological examination and IHC staining

Skin tissues were fixed, embedded in paraffin, and sectioned into 4–5 μm-thick slices. Sections were stained with hematoxylin and eosin (H&E) for histopathological examination. Epidermal thickness was measured at three random sites per section (15 measurements per group) using ImageScope software (Leica Biosystems Imaging, Wetzlar, Germany).

For IHC staining, heat-induced antigen retrieval for the anti-CD11b antibody was performed in citrate buffer (10 mM, pH 6.0) at 60 °C for 1 h. Sections were incubated with Protein Block (0.4% casein in phosphate-buffered saline) for 5 min at room temperature to minimize non-specific binding. Subsequently, sections were incubated overnight at 4 °C with primary antibodies against p65 (1:100 dilution), IL-17A (1:200 dilution), or CD11b (1:1,000 dilution). Following primary incubation, sections were sequentially treated with Post Primary reagent (rabbit anti-mouse IgG), Novolink™ Polymer (anti-rabbit poly-HRP IgG), and 3,3′-diaminobenzidine chromogen according to the manufacturer’s instructions (Leica Biosystems, Wetzlar, Germany). The stained sections were analyzed using ImageJ software (National Institutes of Health, Bethesda, MD, United States). The percentage of stained area was calculated as (brown-stained area/total tissue area) × 100. Additionally, the percentage of stained cells was determined as (number of brown-stained cells/total number of cells) × 100, with 100 cells counted per field. For each section, three non-overlapping fields were randomly selected by an investigator blinded to group allocation.

### 2.5 iTRAQ-based LC-MS/MS and bioinformatic analysis

Total proteins were extracted from skin tissues using ice-cold radioimmunoprecipitation assay lysis buffer (50 mM Tris-HCl, 150 mM NaCl, 1% NP-40, 0.5% sodium deoxycholate, 0.1% sodium dodecyl sulfate, and 1 mM EDTA). Protein concentrations were determined using the Bradford assay (Protein Assay Dye Reagent, Bio-Rad, Hercules, CA, United States). To accommodate sample constraints and focus on exploratory proteomic screening, biological samples from five mice per group were pooled to create a single representative sample per experimental group (mock, IMQ, 200 mg/kg clove, and eugenol).

iTRAQ-based LC-MS/MS was performed as previously described (Liu et al., 2020). Briefly, protein extracts were digested with Trypsin Gold (Promega, Madison, WI, United States) and labeled using iTRAQ 8-plex reagents (Applied Biosystems, Foster City, CA, United States). Labeled peptides were analyzed using an UltiMate 3000 RSLCnano system coupled to an Orbitrap Exploris 480 mass spectrometer (Thermo Fisher Scientific, Waltham, MA, United States). Data processing and protein identification were performed using Proteome Discoverer (version 2.4, Thermo Scientific, Waltham, MA, United States). MS/MS spectra were searched against the UniProt *Mus musculus* database using the following parameters: trypsin as the protease with a maximum of two missed cleavages; precursor mass tolerances of 10 ppm; and fragment mass tolerance of 0.02 Da. Methylthio (C) and iTRAQ 8-plex (K and N-terminus) were defined as static modifications. Variable modifications included oxidation (M) and deamidation (N, Q), along with protein N-terminal modifications (acetylation, Met-loss, and Met-loss + acetylation). Representative MS/MS spectra and unique peptide sequences confirming the identification of IL-36α are provided in Supplementary Figure S1.

Protein quantification was based on the ratio of areas under the iTRAQ reporter ion peaks (117 for mock, 118 for IMQ, 119 for clove, and 121 for eugenol). Normalization was performed at the peptide level, where the intensities of all peptide-to-spectral matches were scaled to the median of the total peptide population to correct for potential loading bias. Final protein-level ratios were calculated as the median of the normalized peptide sequences assigned to each protein. Fold changes were determined by comparing iTRAQ reporter intensities among the experimental groups (IMQ vs. mock, and clove or eugenol vs. IMQ). The statistical significance of protein ratios was determined within Proteome Discoverer, which calculates raw *p*-values for protein ratios based on the distribution and variation of individual peptide ratios assigned to each protein. A one-sample Student’s t-test was applied to the log-transformed ratios to test against a null hypothesis of 1.0 (no change). Proteins were considered differentially expressed if they met a dual criterion of a Benjamini-Hochberg adjusted *p*-value <0.05 and a linear fold change of ≥2 or ≤ −2. This threshold was chosen to minimize false positives and prioritize the most biologically substantial alterations for downstream functional analysis. The mass spectrometry proteomics data have been deposited to the ProteomeXchange Consortium via the PRIDE partner repository with the dataset identifier PXD077433.

Functional annotation and Kyoto Encyclopedia of Genes and Genomes (KEGG) pathway analysis were conducted via the DAVID bioinformatics suite (https://davidbioinformatics.nih.gov/), with significance assessed using the Benjamini-Hochberg adjusted *p*-value <0.05 (false discovery rate (FDR) correction) (Sherman et al., 2022). Heatmaps were subsequently generated using Morpheus (https://software.broadinstitute.org/morpheus).

### 2.6 Competitive IL-17A–IL-17RA binding assay

The competitive binding assay was performed according to the protocol established by Lo et al. (2019). Briefly, 96-well microtiter plates were coated with recombinant human IL-17A (1 ng/well) at 4 °C overnight and incubated at room temperature for 1 h with clove extract (0–40 mg/mL), eugenol (0–10 mM), or recombinant IL-17RA (25 ng/well; positive control). Subsequently, Fc-tagged IL-17RA (50 ng/well) was added for 2 h at 37 °C. Bound IL-17RA was detected using HRP-conjugated goat anti-human IgG Fc antibody (1:5000 dilution) followed by 3,3′,5,5′-tetramethylbenzidine substrate solution (Invitrogen, Camarillo, CA, United States). The reaction was stopped with 2 N H_2_SO_4_, and absorbance was measured at 450 nm using a Multiskan GO microplate reader (Thermo Fisher Scientific, Waltham, MA, United States). The assay was performed in triplicate on two independent occasions. Inhibition was calculated as:

$$\text{Inhibition }\left(\%\right)=\left[1-\frac{\text{absorbance}\;\text{with}\;\text{compounds}}{\text{absorbance}\;\text{without}\;\text{compounds}}\right]\times 100$$

IC_50_ values were calculated by nonlinear regression using a four-parameter logistic curve-fit in Microsoft Excel. The percentage of inhibition was plotted against the log-concentration of the inhibitors, and the IC_50_ was defined as the concentration resulting in a 50% reduction in binding compared to the control.

### 2.7 Molecular docking analysis

Molecular docking of eugenol with IL-17A–IL-17RA complex (PDB ID: 4HSA) was performed using AutoDock Vina 1.1.2 (Trott and Olson, 2010). The eugenol structure was retrieved from PubChem (CID: 3314). Preparation of the protein and ligand was conducted in AutoDock Tools 1.5.7, including the removal of crystallized water molecules, addition of polar hydrogen atoms, and assignment of Kollman charges to the protein. For eugenol, Gasteiger charges were assigned, and all rotatable bonds were kept flexible. Three specific binding regions of IL-17A–IL-17RA interface were defined based on structural hot spots (Liu et al., 2013). Grid boxes were centered at: Region I (−46.657, 61.76, −27.426), Region II (−47.708, 57.69, −33.484), and Region III (−49.448, 29.633, −52.177), with dimensions of 15 × 15 × 15 Å^3^. The exhaustiveness parameter was set to 8. Docking poses were ranked by their binding affinity (kcal/mol); the top-ranked conformation with the lowest binding energy and biologically relevant interactions with key interface residues was selected for further analysis and visualized using PyMOL (Schrödinger Inc., New York, NY, United States).

### 2.8 Statistical analysis

Data are expressed as the mean ± standard error of the mean (SEM). Unless otherwise specified, n represents the number of independent biological replicates. For *in vivo* experiments, n = 5 mice were used per group. For histological and IHC quantification, three random fields or sites were measured per mouse and averaged to provide a single value for each biological replicate. *In vitro* competitive IL-17A–IL-17RA binding assays were conducted on two independent occasions, with each experiment performed in triplicate. For proteomic analysis, a single biological pool was generated for each group by combining equal protein amounts from five individual mice. All statistical comparisons were performed using the biological replicate as the unit of analysis. The normality of data distribution and homogeneity of variance were confirmed using the Shapiro-Wilk test and Levene’s test, respectively, prior to statistical comparison. Statistical significance was determined using One-way ANOVA followed by Tukey’s post-hoc test using SPSS Statistics (IBM, Armonk, NY, United States). A *p*-value <0.05 was considered statistically significant.

## 3 Results

### 3.1 Phytochemical characterization of clove extract

**FIGURE 1:**
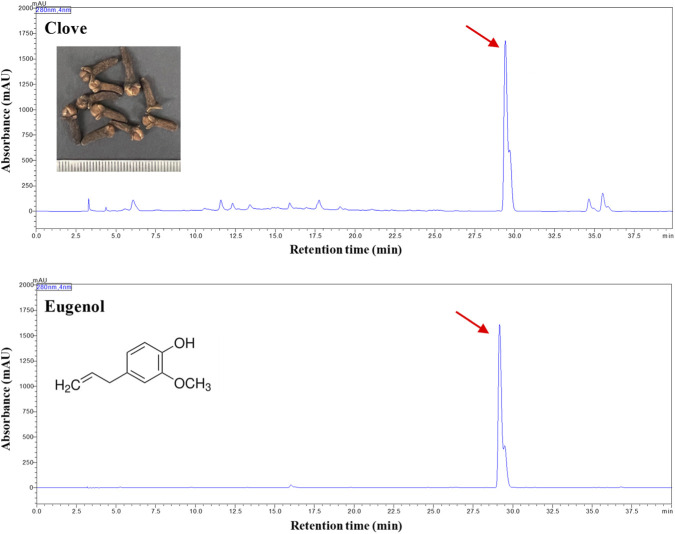
Phytochemical profile of clove extract. HPLC chromatograms of the clove extract (upper panel) and the eugenol standard (lower panel). The arrow indicates the retention time of eugenol in both chromatograms. The dried flower buds of *Syzygium aromaticum* Merr. & L.M. Perry (clove) and the chemical structure of eugenol are shown for reference.

Clove, the dried flower bud of *Syzygium aromaticum* Merr. & L.M. Perry, was analyzed to determine its major phytochemical constituents. Ethanol extracts prepared from standardized clove extract granules were subjected to HPLC analysis. As shown in Figure 1, a dominant peak, representing 65.8% of the total detected phytochemicals was observed at a retention time of 29.422 min. The retention time of the eugenol standard coincided with this peak, confirming that eugenol was the predominant phytochemical compound of the clove extract. The complete set of raw chromatograms and integration parameters s are available in Supplementary Figure S2; Supplementary Table S2.

### 3.2 Clove and eugenol alleviated IMQ-induced psoriasis-like skin lesions

The anti-psoriatic effects of clove and eugenol were evaluated using an IMQ-induced psoriasis-like mouse model. The doses of clove extract (50, 100, and 200 mg/kg) and eugenol (100 mg/kg) were selected based on human-to-animal dose conversion and established murine models. According to the Reagan-Shaw method, a 200 mg/kg dose in mice corresponds to a human equivalent dose of approximately 16.2 mg/kg, which falls within the range of traditional supplemental use (1 g for a 60-kg adult). Furthermore, the 100 mg/kg dose of eugenol has been previously validated as both safe and effective for suppressing systemic inflammation in rodents (Tavvabi-Kashani et al., 2024; Vijayasteltar et al., 2016; Xu et al., 2026), remaining significantly below the oral LD_50_ in rodents (approx. 3,000 mg/kg).

**FIGURE 2:**
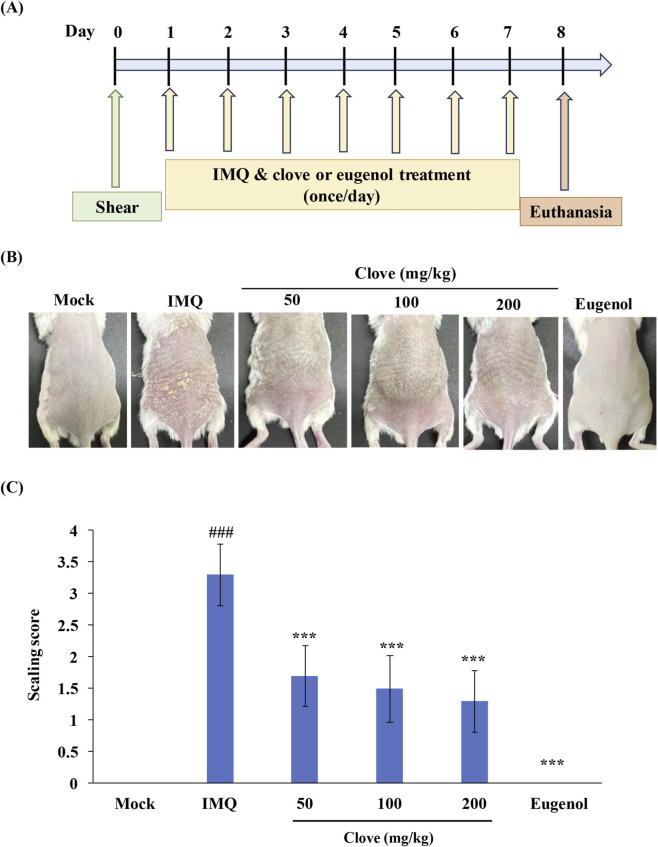
Effects of clove and eugenol on IMQ-induced psoriasis-like skin lesions in mice. Mice were topically treated with Vaseline (mock) or IMQ on the dorsal skin and orally administered various doses (50, 100, or 200 mg/kg) of clove or 100 mg/kg eugenol for seven consecutive days. **(A)** Schematic diagram of the experimental timeline. **(B)** Representative photographs of dorsal skin lesion taken on day 8. **(C)** Scaling scores of dorsal skin lesions were assessed on a scale from 0 to 4. Values are the mean ± SEM of five independent biological replicates. ^###^ *p* < 0.001, compared to the mock group. ^***^ *p* < 0.001, compared to the IMQ group.

Mice with shaved dorsal skin received topical IMQ (62.5 mg/day) and oral administration of clove or eugenol for seven consecutive days (Figure 2A). The severity of psoriatic inflammation was assessed by the psoriasis area and severity index. As shown in Figure 2B, mice in the mock group exhibited smooth and intact dorsal skin, whereas IMQ-treated mice developed pronounced psoriasis-like lesions characterized by erythema, thickening, and scaling, resembling human plaque psoriasis. Oral administration of clove or eugenol markedly alleviated these skin lesions, with eugenol producing a more pronounced effect. Consistently, scaling scores were significantly reduced in both treatment groups compared with the IMQ group (Figure 2C). The health and wellbeing of the mice were monitored daily throughout the study. No clinical signs of systemic toxicity, such as lethargy or significant changes in food and water intake, were observed. Furthermore, there were no significant differences in body weight between the treatment groups and the control group at the end of the 7-day period, suggesting that the oral doses were well-tolerated.

**FIGURE 3:**
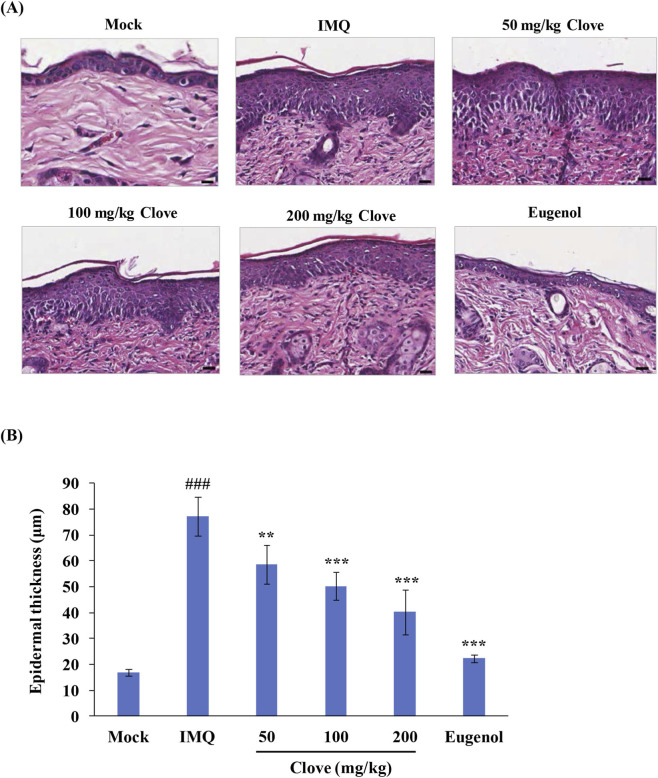
Effect of clove and eugenol on IMQ-induced histopathological changes in mouse skin. Mice were treated daily with Vaseline cream (mock) or IMQ on the shaved dorsal skin for seven consecutive days. In the clove and eugenol groups, mice received topical IMQ application and oral administration of clove (50, 100, or 200 mg/kg) or eugenol (100 mg/kg). **(A)** Representative H&E-stained skin sections (original magnification of 200×). Scale bar = 10 μm. **(B)** Quantification of epidermal thickness. Values represent the mean ± SEM of five independent biological replicates (n = 5 mice per group). For each mouse, measurements were averaged from three distinct sites to provide a representative value. ^###^ *p* < 0.001, compared to the mock group. ^**^ *p* < 0.01, ^***^ *p* < 0.001, compared to the IMQ group.

Histopathological examination further confirmed the protective effects of clove and eugenol. H&E staining revealed typical psoriasiform changes in the IMQ group, including hyperkeratosis and epidermal hyperplasia (Figure 3A). Epidermal thickness was significantly increased in the IMQ group (77.18 ± 7.61 μm) compared with the mock group (16.95 ± 1.43 μm) (Figure 3B). Treatment with clove reduced epidermal thickness to 58.59 ± 7.59, 50.25 ± 5.38, and 40.22 ± 8.56 μm at doses of 50, 100, and 200 mg/kg, respectively, while eugenol (100 mg/kg) reduced epidermal thickness to 22.36 ± 1.59 μm. Additionally, the inhibitory effect of clove on epidermal hyperplasia was dose-dependent.

### 3.3 Proteomic alterations induced by clove and eugenol

To elucidate the molecular mechanisms underlying the anti-psoriatic effects of clove and eugenol, exploratory quantitative proteomic profiling was performed using iTRAQ-based LC-MS/MS on pooled skin tissue samples (n = 5 per pool) from the mock, IMQ, clove (200 mg/kg), and eugenol (100 mg/kg) groups. A total of 65,899 peptides were identified with a FDR below 1%, corresponding to 7,416 confidently identified proteins (≥1 peptide, >95% confidence).

**FIGURE 4:**
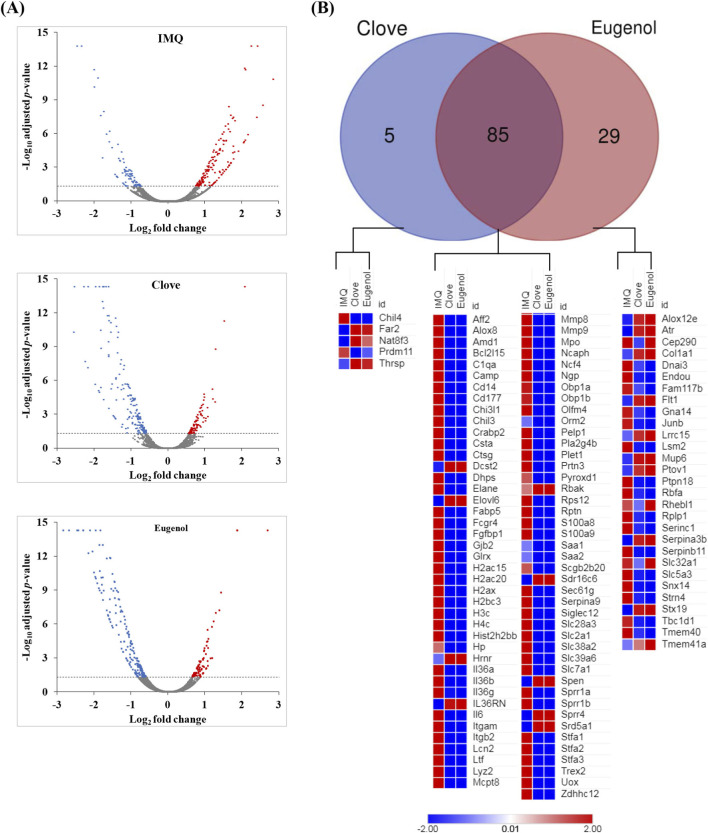
Proteomic alterations in psoriatic-like skin following clove and eugenol treatment. **(A)** Volcano plots showing the global proteomic changes in skin tissues of IMQ-, clove extract-, and eugenol-treated mice. Red dots indicate proteins with a fold change ≥2 (log_2_ fold change ≥1) and statistically significant differences (adjusted *p* < 0.05), while blue dots represent significantly downregulated proteins (fold change ≤ −2 (log_2_ fold change ≤ −1), adjusted *p* < 0.05). Gray dots indicate proteins without statistically significant changes. The dot line denotes significant threshold. **(B)** Venn diagram illustrating the overlap of proteins (fold change ≥2 or ≤ −2) altered by clove and eugenol treatments. Heatmaps below showing the expression patterns of proteins affected by IMQ, clove, or eugenol. Fold changes are color-coded according to the scale shown below, with increased protein levels in red and decreased levels in blue.

To visualize global proteomic alterations among treatment groups, volcano plots were generated to display the relationship between the statistical significance (−Log_10_ adjusted *p*-value) and the magnitude of change (Log_2_ fold change) for each protein. Compared with the mock group, IMQ treatment markedly altered the skin proteome, with a large number of proteins being upregulated (Figure 4A). In contrast, treatment with clove or eugenol reversed many of these alterations, primarily by downregulating proteins induced by IMQ.

### 3.4 Clove and eugenol modulated the IL-36 signaling axis

Proteins with fold changes ≥2 or ≤ −2 and adjusted *p*-values <0.05 were selected for further analysis. Relative to the mock group, IMQ upregulated 130 proteins and downregulated 56 proteins. Compared with the IMQ group, clove treatment upregulated 11 proteins and downregulated 79 proteins, whereas eugenol upregulated 20 proteins and downregulated 94 proteins. Venn diagram analysis was performed to compare the similarity of proteins affected by clove and eugenol. As shown in Figure 4B, 85 proteins were commonly regulated by both clove and eugenol, while 5 and 29 proteins were uniquely affected by clove and eugenol, respectively. Heatmap visualization showed that the expression patterns of the commonly regulated proteins were reversed by treatment. Proteins upregulated by IMQ were downregulated following clove and eugenol treatment. The high similarity in proteomic profiles between clove and eugenol suggested that eugenol represented a major bioactive component responsible for the anti-psoriatic activity of clove.

Proteins commonly regulated by both clove and eugenol were grouped according to their functional similarity using DAVID (Table 1). Functional annotation clustering revealed significant enrichment of IL-36-related proteins (Group Enrichment Score >1.3). IMQ treatment increased the expression of IL-36α, IL-36β, and IL-36γ while reducing IL-36 receptor antagonist (IL-36Ra). Both clove and eugenol reversed these changes, decreasing IL-36 cytokines and increasing IL-36Ra levels. Given that IL-36 signaling functions as a key amplifier of psoriatic inflammation, these results suggested that clove and eugenol alleviated psoriasis-like lesions in part through modulation of the IL-36 signal axis.

**TABLE 1:** Functional classification of proteins commonly affected by both clove and eugenol.

Accession	Gene symbol	Protein	FC_IMQ	FC_Clove	FC_Eugenol	Adjusted *p* value_IMQ	Adjusted *p* value_Clove	Adjusted *p* value_Eugenol	Coverage (%)	# Peptides
Group 1 enrichment score: 17.071										
Q64523	H2ac20	Histone H2A type 2-C	3.538	−3.175	−2.882	7.2E-08	4.74E-15	8.08E-10	36	6
P27661	H2ax	Histone H2AX	3.004	−2.833	−2.924	0.0022	2.04E-06	6.93E-07	43	8
Q8CGP7	H2ac15	Histone H2A	2.81	−2.653	−2.695	3.42E-05	2.93E-12	5.29E-09	41	7
P62806	H4c	Histone H4	2.747	−2.771	−2.976	0.0046	0.0013	3.92E-07	53	9
P68433	H3c1	Histone H3.1	2.824	−2.882	−2.841	0.0032	5.48E-06	1.47E-06	62	9
Q64525	Hist2h2bb	Histone H2B type 2-B	3.159	−3.031	−2.924	4E-09	4.74E-15	1.57E-12	63	10
Q64475	H2bc3	Histone H2B type 1-B	2.984	−2.725	−2.801	0.0016	0.0016	2.12E-06	63	10
Group 2 enrichment score: 4.821										
P56567	Csta	Cystatin-A	2.751	−2.188	−2.309	0.0046	0.0181	0.0003	90	8
P35173	Stfa3	Stefin-3	5.979	−3.012	−3.636	2.98E-09	0.0003	7.09E-10	82	13
P35174	Stfa2	Stefin-2	4.809	−4.444	−5.348	1.61E-14	4.74E-15	5.38E-15	47	4
P35175	Stfa1	Stefin-1	4.521	−2.674	−2.976	1.24E-06	0.0021	4.14E-07	64	8
Group 3 enrichment score: 3.768										
P43430	Mcpt8	Mast cell protease 8	2.485	−2.283	−2.262	0.0002	4.46E-05	3.74E-05	4	1
Q3UP87	Elane	Neutrophil elastase	2.383	−2.519	−2.703	2.96E-05	5.11E-11	2.01E-10	8	2
Q61096	Prtn3	Myeloblastin	5.405	−5.747	−6.369	1.61E-14	4.74E-15	5.38E-15	5	1
P28293	Ctsg	Cathepsin G	3.4	−3.367	−3.436	5.1E-05	4.74E-15	3.5E-10	15	4
Group 4 enrichment score: 3.635										
Q9JLA2	Il36α	Interleukin-36 alpha	4.247	−3.861	−4.292	1.49E-12	4.74E-15	5.38E-15	31	4
Q8R460	Il36γ	Interleukin-36 gamma	2.295	−1.984	−2.222	0.0001	1.98E-05	4.38E-07	19	2
Q9D6Z6	Il36β	Interleukin-36 beta	2.168	−2.004	−2.179	0.0008	4.46E-05	2.84E-06	15	4
Q9QYY1	IL36Ra	Interleukin-36 receptor antagonist protein	−2.371	1.984	2.114	4.3E-05	1.63E-05	3.38E-06	29	3

### 3.5 Clove and eugenol regulated the IL-17 signaling pathway

**FIGURE 5:**
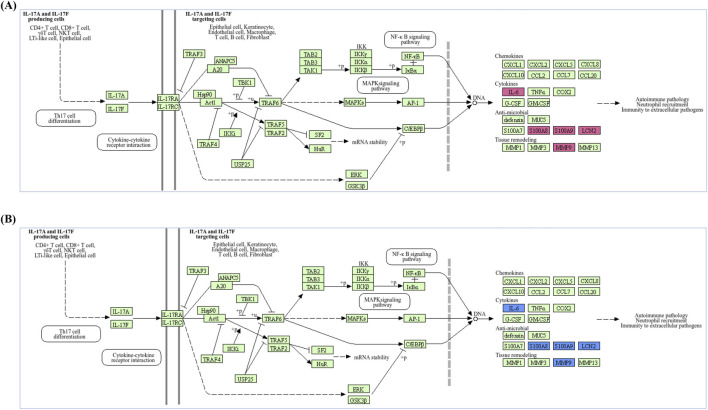
KEGG pathway maps affected by IMQ and clove/eugenol treatments. **(A)** IMQ treatment. **(B)** Clove and eugenol treatment. Red frames indicate upregulated proteins (fold change ≥2), and blue frames indicate downregulated proteins (fold change ≤ −2).

To further identify affected biological pathways, KEGG pathway analysis was performed on proteins commonly regulated by clove and eugenol. As shown in Table 2, a total 16 pathways were significantly enriched. Among them, several immune-related pathways were identified, including neutrophil extracellular trap formation, systemic lupus erythematosus, phagosome, leukocyte transendothelial migration, and the IL-17 signaling pathway. Within the IL-17 signaling pathway, IMQ treatment increased the expression of inflammatory mediators, including IL-6, anti-microbial proteins (S100A8, S100A9, and lipocalin-2), and matrix metalloproteinase-9 (MMP-9) (Figure 5). Treatment with clove or eugenol suppressed the expression of these IMQ-induced proteins.

**TABLE 2:** KEGG analysis of proteins commonly altered by both clove and eugenol.

Term	Count^a^	Involved proteins/total proteins (%)	Adjusted *p-*value
Neutrophil extracellular trap formation	30	27.027	1.20E-31
Systemic lupus erythematosus	26	23.423	3.66E-29
Alcoholism	22	19.819	1.00E-19
Viral carcinogenesis	15	13.514	2.67E-10
Coronavirus disease - COVID-19	13	11.712	2.05E-06
Ribosome	11	9.909	1.05E-05
Transcriptional misregulation in cancer	10	9.009	1.98E-05
Phagosome	7	6.306	0.0015
Pertussis	5	4.505	0.0018
IL-17 signaling pathway	5	4.505	0.0036
Amoebiasis	5	4.505	0.0061
Tuberculosis	6	5.405	0.0074
Legionellosis	4	3.604	0.0078
*Staphylococcus aureus* infection	5	4.505	0.0111
Leishmaniasis	4	3.604	0.0119
Leukocyte transendothelial migration	4	3.604	0.0469

^a^ Number of altered proteins in this term.

**FIGURE 6:**
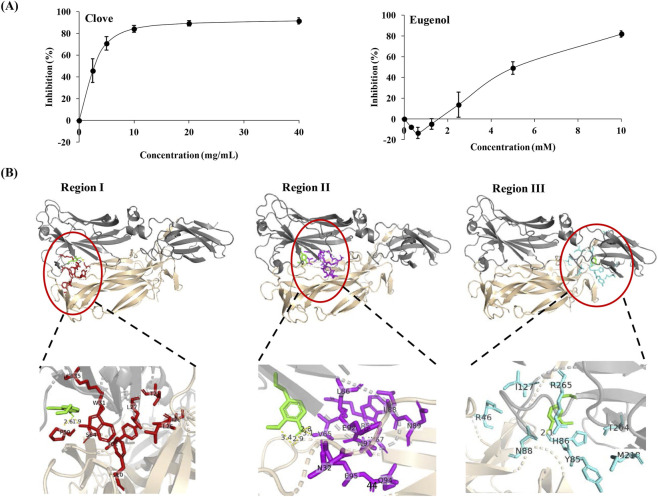
Effects of clove and eugenol on IL-17A–IL-17RA interaction. **(A)** Competitive IL-17A–IL-17RA binding assay. IL-17A-coated microplates were incubated with various concentrations of clove (left panel) or eugenol (right panel). After incubation for 1 h at room temperature, Fc-tagged IL-17RA, HRP-conjugated goat anti-human IgG Fc antibody, and chromogenic substrate were sequentially added. Data are presented as inhibition (%). Values represent the mean ± SEM of two independent experiments, each performed in triplicate. **(B)** Docking structure of eugenol (green) with the IL-17A (wheat)–IL-17RA (gray) complex. The crystal structure (PDB ID: 4HSA) of IL-17A in the complex with IL-17RA was obtained from Protein Data Bank. Critical amino acid residues located in Region I, II, and III involved in the IL-17A–IL-17RA interaction are shown in red, pink, and cyan, respectively. Enlarged pictures show the amino acid residues forming interactions between eugenol and the IL-17A–IL-17RA complex. Yellow dashed lines represent hydrogen bonds between eugenol and the IL-17A–IL-17RA complex.

Because IL-17 signaling is initiated by binding of IL-17A to its receptor IL-17RA, a competitive IL-17A–IL-17RA binding assay was performed to determine whether clove and eugenol interfered with this interaction. As shown in Figure 6A, both clove and eugenol inhibited IL-17A–IL-17RA binding in a concentration-dependent manner, with IC_50_ values of 2.97 ± 0.8 mg/mL and 5.2 ± 0.7 mM, respectively. To further explore the molecular basis of this interaction, *in silico* docking analysis was performed using the crystal structure of the IL-17A–IL-17RA complex (PDB: 4HSA). The critical residues mediating IL-17A–IL-17RA binding were classified into three regions, as reported in the X-ray crystal structure (Liu et al., 2013). Eugenol exhibited binding affinities of −5.7, −4.2, and −4.1 kcal/mol at Regions I, II, and III of the interface, respectively. These negative values indicated thermodynamically favorable binding at all three targeted sites. Notably, given that eugenol is a small, low-molecular-weight compound (164.2 g/mol), these values represent significant binding relative to its molecular size. Detailed interaction analysis revealed that eugenol formed key hydrogen bonds with Ser64 and Val65 of the IL-17A B-chain and His86 of the A-chain (Figure 6B). Collectively, these docking insights and competitive binding data suggested that clove and eugenol may physically disrupt the IL-17A–IL-17RA interface, thereby attenuating downstream signaling.

### 3.6 Clove and eugenol inhibited NF-κB activation, IL-17A production, and granulocyte infiltration

**FIGURE 7:**
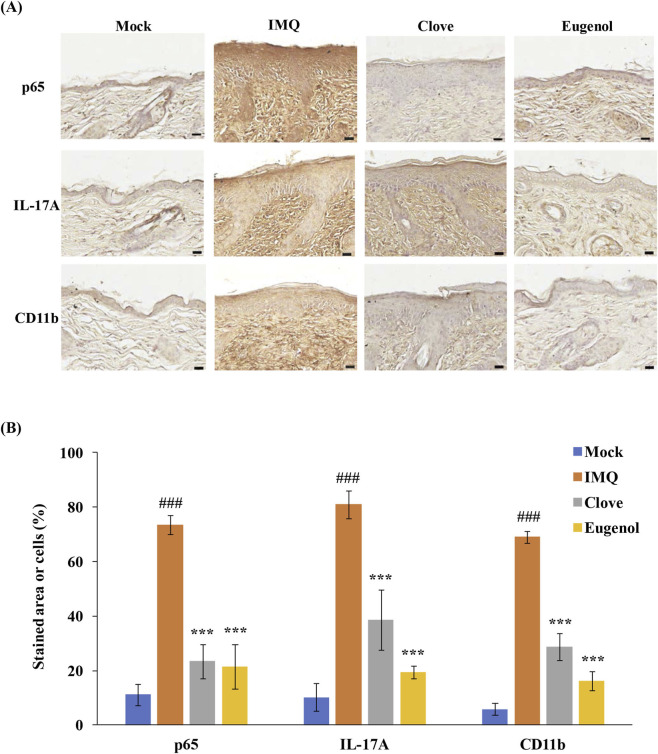
Effects of clove and eugenol on NF-κB activation, IL-17A expression, and granulocyte infiltration in IMQ-induced psoriatic-like skin lesions. IHC staining of dorsal skin tissues was performed using antibodies against total p65, IL-17A, and CD11b. **(A)** Representative images (original magnification of 200×). Scale bar = 10 μm. **(B)** Quantification of stained area or cells (%). Data are presented as the mean ± SEM of five independent biological replicates (n = 5 mice per group). For each mouse, measurements were averaged from three distinct fields to provide a representative value. ^###^ *p* < 0.001, compared to the mock group. ^***^ *p* < 0.001, compared to the IMQ group.

IL-17A–IL-17RA interaction activates the NF-κB pathway, leading to cytokine release and immune cell infiltration. Therefore, the effects of clove and eugenol on NF-κB signaling were examined by IHC staining for p65, IL-17A, and CD11b. As shown in Figure 7, IMQ treatment markedly increased nuclear translocation of p65, suggesting the induction of NF-κB activation. IMQ also enhanced production of IL-17A protein and promoted infiltration of CD11b-positive cells in skin tissues. Treatment with clove or eugenol significantly reduced p65 nuclear translocation, decreased IL-17A production, and suppressed granulocyte infiltration. These findings suggested that clove and eugenol suppressed IL-17A–IL-17RA signaling and downstream NF-κB activation, thereby reducing IL-17A production and granulocyte infiltration, ultimately contributing to the amelioration of psoriasis-like skin inflammation.

## 4 Discussion

Medicinal herbs traditionally used to treat inflammatory skin disorders have increasingly been recognized as promising sources of anti-psoriatic therapies (Zhou et al., 2026). Recent studies have shown that several ethnomedicinal formulations and phytochemicals, including burdock (*Arctium lappa*), indigo naturalis, Fufang Longdan Mixture, licoisoflavone B from licorice, triptolide from *Tripterygium wilfordii*, and phenolic compounds such as vanillin, ferulic acid, and rosmarinic acid, can attenuate psoriasiform inflammation through diverse immunomodulatory mechanisms. These mechanisms include suppression of Th17/IL-17-mediated inflammation, modulation of the Wnt5a/β-catenin signaling pathway, and inhibition of NF-κB, JAK/STAT, and NLRP3 inflammasome signaling pathways, as well as the miR-29a-5p/insulin-like growth factor 1 receptor axis (Chen et al., 2025; Cheng et al., 2017; Ho et al., 2022; Liu et al., 2026b; Lo et al., 2019; Luo et al., 2025; Yang et al., 2025; Zhao et al., 2025). Several studies have also highlighted the expanding role of natural compounds in modulating immune responses, particularly in the context of chronic inflammatory conditions (Wang et al., 2024b; Wang et al., 2025b). Against this background, clove, a medicinal plant long used in Asia, Africa, and the Middle East for the treatment of wounds, inflammation, and skin disorders, has not been well explored for its potential role in psoriasis. In the present study, we demonstrate that oral administration of clove extract ameliorates IMQ-induced psoriasiform skin lesions, thereby providing pharmacological support for its traditional dermatological use.

Clove is rich in essential oils, comprising approximately 15%–20% of the dried bud, with eugenol representing the predominant component and accounting for 70%–95% of the oil fraction (Haro-González et al., 2021). In the standardized concentrated clove extract granules used in this study, eugenol represented 65.8% of the detected phytochemical composition. Consistently, proteomic analyses revealed a substantial overlap in the molecular responses induced by clove and eugenol, suggesting that eugenol was likely a major contributor to the observed anti-inflammatory effects. Eugenol is a multifunctional phytochemical with documented antimicrobial, antioxidant, and anti-inflammatory activities (Lao et al., 2024; Tavvabi-Kashani et al., 2024). While recent studies have shown that topical eugenol improves IMQ-induced psoriasis by modulating IL-17A, IL-1β, IL-6, IL-23, and tumor necrosis factor-α (TNF-α), as well as JAK1/STAT3 signaling and oxidative stress pathways (Guo et al., 2025; Keshari et al., 2024), our findings demonstrate that orally administered eugenol also exerts significant therapeutic effects. As a small, lipophilic molecule (log*P* of approximately 2.27), eugenol facilitates efficient passive diffusion across biological membranes (Guenette et al., 2006; Reiner et al., 2009). This suggests that gastrointestinal absorption and subsequent systemic distribution allow clove-derived phytochemicals to regulate immune responses beyond the local epidermal environment. Collectively, these findings support the role of clove and eugenol as potent modulators of the systemic inflammatory pathways associated with psoriasis.

A key finding in the present study was the regulatory effect of clove and eugenol on the IL-36 cytokine family. IL-36α, IL-36β, and IL-36γ are markedly upregulated in psoriatic lesions and act synergistically with IL-17A and TNF-α to amplify inflammatory signaling, promote keratinocyte hyperactivation, and recruit neutrophils (Sachen et al., 2022). In contrast, IL-36Ra functions as a natural antagonist that competitively inhibits IL-36 receptor activation. Dysregulation of IL-36 signaling is particularly associated with severe forms of psoriasis and pharmacological targeting of IL-36 receptor has recently emerged as a promising therapeutic strategy (Okorie et al., 2024; Vilaça et al., 2024). In this study, oral administration of clove or eugenol significantly reduced the expression of IL-36 agonists while increasing IL-36Ra levels. Restoration of this agonist–antagonist balance may help attenuate IL-36-driven inflammatory signaling and thereby reduce downstream activation of psoriasis-associated cytokine networks.

Consistent with the modulation of IL-36 signaling, treatment with clove extract or eugenol significantly reduced IL-17A expression in skin tissues. In the context of psoriasis, IL-36 and IL-17A are not linked in a simple linear cascade; rather, they form a reciprocal amplification loop that drives chronic inflammation. IL-36 cytokines promote the induction of the Th17 response, leading to increased IL-17A production, while IL-17A synergizes with IL-36 to enhance the expression of pro-inflammatory chemokines and antimicrobial peptides in keratinocytes (Pfaff et al., 2017). Our results suggest that clove extract and eugenol disrupt this circuit by targeting the IL-17A/IL-17RA interface. The physical interference at this interface, predicted by our docking models, is consistent with the reduced localized IL-17A protein levels observed via IHC. Because IL-17A is a potent driver of IL-36 expression, the inhibition of the IL-17A node leads to a secondary suppression of the IL-36 axis. This is evidenced by our proteomic findings, which show a significant downregulation of the IL-36-responsive S100A8 and S100A9. Collectively, these data support a model where eugenol attenuates psoriasiform pathology by collapsing the reciprocal IL-17A/IL-36 signaling loop. Although IL-17A was not detected in the proteomic dataset, this is a common occurrence in discovery-based proteomics due to the extreme dynamic range of the skin proteome. High-abundance structural proteins, such as keratins, frequently mask low-abundance cytokines during LC-MS/MS analysis. To address this, we utilized IHC as a sensitive and spatially resolved validation method. IHC is optimized for detecting localized cytokine signals that might be diluted or lost during the homogenization required for proteomic workflows. The significant reduction in IL-17A protein levels observed via IHC, coupled with the proteomic downregulation of downstream IL-17A effectors (e.g., S100A8, S100A9, and MMP-9), provides cohesive evidence that clove extract and eugenol effectively suppress the IL-17A signaling axis in psoriatic skin.

Additional evidence for the involvement of the IL-17 pathway was provided by competitive binding assays, which confirmed that eugenol inhibited the IL-17A–IL-17RA interaction with an IC_50_ of 5.2 mM. These findings suggest that eugenol may interfere directly with cytokine–receptor binding. To provide context, we benchmarked this potency against other phenylpropanoids from our previous screening (Ho et al., 2022), where rosmarinic acid (2.14 ± 0.35 mM), syringic acid (4.79 ± 0.2 mM), and eugenol (5.35 ± 0.1 mM) emerged as the most effective candidates. This comparison identifies eugenol as a top-tier lead within its chemical class. While these millimolar (mM) values are higher than the nanomolar (nM) potencies of biologics such as Secukinumab (∼0.3 nM) and Bimekizumab (∼0.003 nM) (Liu et al., 2026a), it is important to note that disrupting large, flat protein-protein interfaces with small molecules is a significant challenge. Given the low molecular weight of eugenol (164.2 g/mol) compared to monoclonal antibodies (∼150 kDa), these results represent high ligand efficiency and provide a viable structural scaffold for future medicinal chemistry optimization. To provide a structural rationale for this observed inhibition, docking simulations were performed using the crystal structure of the IL-17A–IL-17RA complex (PDB: 4HSA). Since this structure captures a protein-protein interface lacking a bound small-molecule inhibitor, a traditional root-mean-square deviation-based re-docking validation was not feasible. Therefore, we employed a residue-specific validation strategy, benchmarking our results against the interface ‘hot spots’ identified by Liu et al. (2013). The predicted docking poses for eugenol demonstrated significant spatial overlap with the binding footprint of IL-17A, specifically involving key residues in Regions I, II, and III. Notably, eugenol interacted with Val-65 on the B-chain and His-86 on the A-chain; Val-65 is an essential contact residue for IL-17RA engagement (Liu et al., 2013), and steric interference at this position is likely to weaken receptor recruitment. This high spatial correlation between eugenol’s binding modes and biologically critical residues supports the structural relevance of our model. While these findings establish a clear inhibitory role, further biophysical characterization using surface plasmon resonance or isothermal titration calorimetry will be required to definitively determine the binding kinetics and thermodynamic parameters of this interaction.

NF-κB signaling plays a critical role in the regulation of inflammation (Du et al., 2026; Jiang et al., 2025; Wang et al., 2025a). In psoriasis, the NF-κB signaling is a central downstream pathway activated by both IL-36 and IL-17 signaling cascades (Griffiths et al., 2021). In the present study, reduced p65 immunostaining was observed following treatment with clove extract or eugenol, suggesting suppression of NF-κB activation. Because NF-κB regulates the expression of numerous pro-inflammatory mediators involved in keratinocyte proliferation and immune cell recruitment, inhibition of this pathway likely contributes to the overall improvement in psoriasiform skin pathology observed in the treated animals. Collectively, these findings suggest that clove-derived phytochemicals modulate multiple interconnected inflammatory pathways, including IL-36, IL-17, and NF-κB signaling, thereby mitigating psoriasiform inflammation in this experimental model.

The systemic safety of clove and eugenol is an essential consideration for their potential use as long-term adjunctive therapies. Clove oil is classified as Generally Recognized as Safe by the U.S. Food and Drug Administration, and eugenol has been evaluated as safe by international regulatory agencies, with a lethal dose at 50% of approximately 2,650-3,000 mg/kg in rats. Eugenol is rapidly metabolized and eliminated, supporting its suitability for oral administration (Tavvabi-Kashani et al., 2024; Vijayasteltar et al., 2016). In the present study, no overt toxicity was observed in mice treated with clove extract or eugenol at the tested doses. Nevertheless, additional studies examining pharmacokinetics and long-term safety will be necessary to further define the translational potential of clove-derived bioactives.

Despite the insights provided by this study, several limitations should be acknowledged. First, although the IMQ-induced model is a standard surrogate for human psoriasis, the current study is limited by the absence of human cell validation. Future research involving human primary keratinocytes and correlation with clinical samples will be essential to further validate the therapeutic potential of eugenol. Second, the proteomic analysis was performed using pooled samples without individual biological replicates, which limits the statistical power and robustness of these specific findings. However, this exploratory approach was utilized primarily for hypothesis generation, and key protein changes were subsequently validated using independent biological replicates through IHC, ensuring the biological relevance of the identified pathways. Third, the current study did not include a standard-of-care positive control, such as methotrexate or a clinical IL-17 inhibitor. While our results demonstrate the anti-inflammatory and IL-17A-inhibitory properties of clove extract and eugenol, further studies are required to directly compare their efficacy and safety profiles against established pharmaceutical agents. Nonetheless, the direct inhibition of the IL-17A/IL-17RA interaction observed here suggests that these natural compounds may serve as promising, low-toxicity adjunctive therapies for psoriasis management.

In conclusion, the present study demonstrates that oral administration of clove extract and its major bioactive constituent, eugenol, significantly attenuates IMQ-induced psoriasiform skin inflammation in mice. These protective effects are associated with modulation of key inflammatory pathways, including restoration of IL-36 cytokine balance, suppression of IL-17A expression, and inhibition of downstream NF-κB activation, ultimately reducing keratinocyte hyperproliferation and inflammatory cell infiltration. Molecular docking analysis further suggested that eugenol may exert these effects by directly interacting with key residues at the IL-17A/IL-17RA interface, providing a structural rationale for its inhibitory activity. Collectively, these findings elucidate the multi-target immunomodulatory mechanisms underlying the anti-inflammatory effects of clove and highlight the capacity of eugenol to regulate immune pathways involved in chronic inflammatory skin disorders. Notably, in conjunction with its favorable oral bioavailability, safety profile, and longstanding ethnomedicinal use, this study offers pharmacological support for the traditional application of clove in skin inflammation. However, further studies, including clinical investigations, are warranted to validate its efficacy and safety in humans and to fully establish its potential as an adjunctive therapeutic for psoriasis and related inflammatory dermatoses.

## Data Availability

The datasets presented in this study can be found in online repositories. The names of the repository/repositories and accession number(s) can be found below: PRIDE Archive, PXD077433 (https://www.ebi.ac.uk/pride/archive/projects/PXD077433).
