# Supplementary material for: Clove (Syzygium aromaticum) and its bioactive constituent eugenol alleviate psoriatic inflammation by modulating the IL-36/IL-17A axis: a proteomic and mechanistic study
Source: Front Pharmacol. 2026 May 26;17:1839501. doi: 10.3389/fphar.2026.1839501 (PMC13246417; doi:10.3389/fphar.2026.1839501)
Supplement: Supplementary file 1 [file Supplementaryfile1.docx]

**Supplementary TABLE S1 Detailed information of the antibodies used in this study.**

| **Antibody** | **Description** | **Company** | **Catalog No.** | **Assay** | **Dilution** | **Antigen retrieval** |
| --- | --- | --- | --- | --- | --- | --- |
| Anti-p65 | Mouse monoclonal (12H11) to NF-κB (p65 subunit) | Millipore | MAB3026 | IF  IHC-P  IHC-Fr  WB (ECL)  Supershift assay | 1:100-1:1,000  1:100-1:200  1:100-1:200  1:100-1:200  1:1,000-1:2,000 | No |
| Anti-IL-17A | Rabbit polyclonal to IL-17A | Bioss | bs-1183R | IHC-P  IF | 1:200-1:400  1:50-1:200 | No |
| Anti-CD11b | Rabbit monoclonal (EPR1344) to CD11b | Abcam | ab133357 | WB  IHC-P | 1:1,000  1:1,000-1:4,000 | Citrate buffer (pH 6.0) at 60 °C |
| Goat anti-human IgG Fc | Whole molecule, absorbed for dual labeling or F(ab’)_2_ fragment affinity purified antibody, HRP conjugated | Millipore | AP113P | WB (chromogenic)  WB (ECL)  IHC/ICC  ELISA (chromogenic) | 1:5,000-1:100,000  1:10,000-1:200,000  1:500-1:5,000  1:5,000-1:100,000 | No |

**Supplementary TABLE S2 HPLC integration reports of clove extract and eugenol.**

**(A) Clove extract**

| **Peak#** | **Retention Time** | **Height** | **Peak area** | **Area%** |
| --- | --- | --- | --- | --- |
| 1 | 3.296 | 123951 | 568580 | 1.213 |
| 2 | 3.691 | 2810 | 8778 | 0.019 |
| 3 | 4.378 | 40228 | 199896 | 0.427 |
| 4 | 5.575 | 16905 | 264717 | 0.565 |
| 5 | 6.077 | 105806 | 1773779 | 3.785 |
| 6 | 10.578 | 21088 | 258537 | 0.552 |
| 7 | 10.805 | 11944 | 160967 | 0.344 |
| 8 | 11.582 | 100377 | 1161832 | 2.479 |
| 9 | 12.316 | 66160 | 790150 | 1.686 |
| 10 | 12.667 | 10936 | 136663 | 0.292 |
| 11 | 13.41 | 46255 | 635949 | 1.357 |
| 12 | 13.76 | 3769 | 25857 | 0.055 |
| 13 | 14.514 | 5420 | 47093 | 0.1 |
| 14 | 14.934 | 15821 | 271265 | 0.579 |
| 15 | 15.154 | 17744 | 203442 | 0.434 |
| 16 | 15.403 | 3531 | 17179 | 0.037 |
| 17 | 15.898 | 57768 | 676401 | 1.443 |
| 18 | 16.288 | 10564 | 143081 | 0.305 |
| 19 | 16.587 | 6736 | 68317 | 0.146 |
| 20 | 16.952 | 13266 | 185108 | 0.395 |
| 21 | 17.74 | 88753 | 1270313 | 2.711 |
| 22 | 18.88 | 6139 | 42170 | 0.09 |
| 23 | 19.058 | 31301 | 333600 | 0.712 |
| 24 | 19.364 | 13727 | 141214 | 0.301 |
| 25 | 21.037 | 10103 | 113337 | 0.242 |
| 26 | 22.293 | 6716 | 52397 | 0.112 |
| 27 | 23.421 | 4788 | 40366 | 0.086 |
| 28 | 24.68 | 7125 | 71230 | 0.152 |
| 29 | 24.939 | 8929 | 98140 | 0.209 |
| 30 | 25.155 | 9545 | 101029 | 0.216 |
| 31 | 25.387 | 7263 | 94335 | 0.201 |
| 32 | 28.928 | 4775 | 61649 | 0.132 |
| 33 | 29.422 | 1681067 | 24222150 | 51.69 |
| 34 | 29.695 | 601496 | 6594615 | 14.073 |
| 35 | 34.659 | 123104 | 1590793 | 3.395 |
| 36 | 34.965 | 31987 | 386596 | 0.825 |
| 37 | 35.526 | 178634 | 2485806 | 5.305 |
| 38 | 35.857 | 46046 | 613577 | 1.309 |
| 39 | 42.524 | 6779 | 59266 | 0.126 |
| 40 | 45.889 | 52823 | 644009 | 1.374 |
| 41 | 47.936 | 20966 | 218448 | 0.466 |
| 42 | 49.401 | 4083 | 27542 | 0.059 |

**(B) Eugenol**

| **Peak#** | **Retention Time** | **Height** | **Peak area** | **Area%** |
| --- | --- | --- | --- | --- |
| 1 | 3.473 | 22347 | 53236 | 0.19 |
| 2 | 15.773 | 24654 | 346266 | 1.233 |
| 3 | 29.032 | 1514642 | 20633865 | 73.482 |
| 4 | 29.287 | 565318 | 6334971 | 22.56 |
| 5 | 36.625 | 5664 | 53757 | 0.191 |
| 6 | 45.808 | 38675 | 471659 | 1.68 |
| 7 | 47.882 | 16886 | 186509 | 0.664 |

**Supplementary FIGURE S1.** Representative LC-MS/MS spectra of peptides corresponding to IL-36α identified in the proteomic analysis. Four unique peptides with high confidence are shown, confirming the identification of IL-36α.


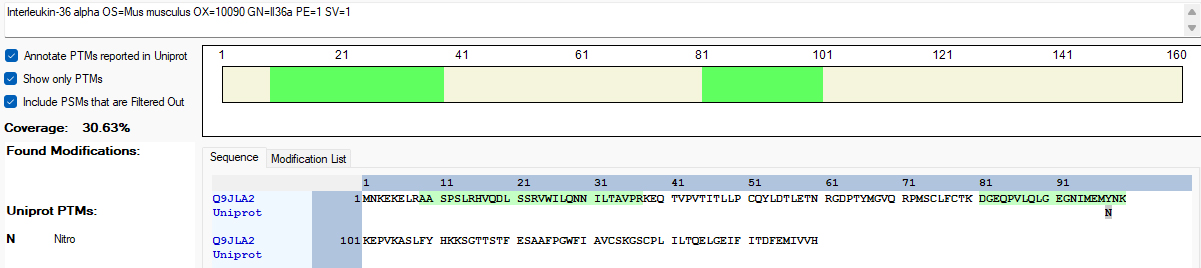


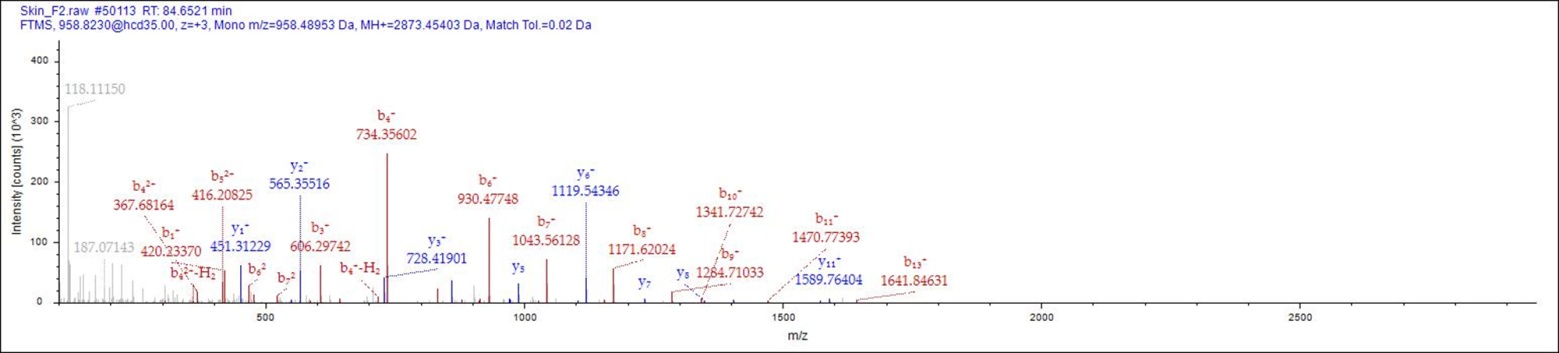


K.DGEQPVLQLGENIMEMYNK.K at m/z 958.48953


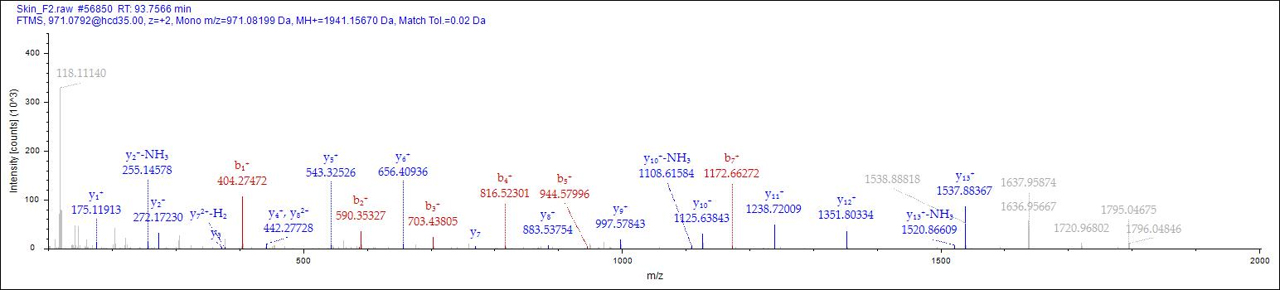


R.VWILQNNILTAVPR.K at m/z 971.08199


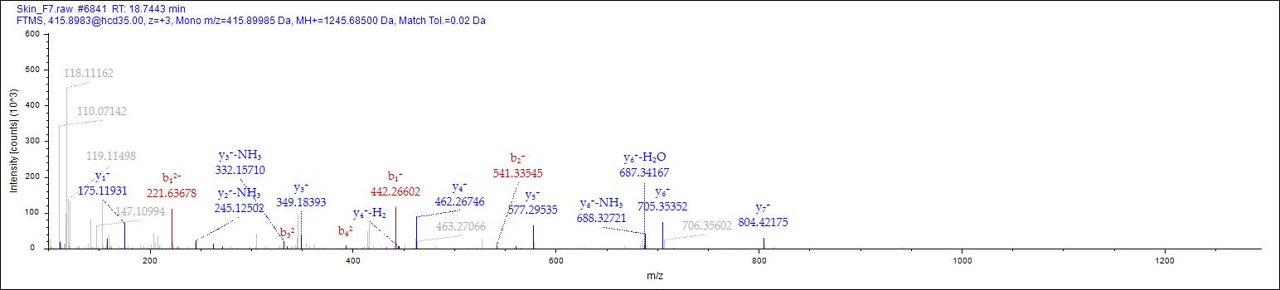


R.HVQDLSSR.V at m/z 415.89985


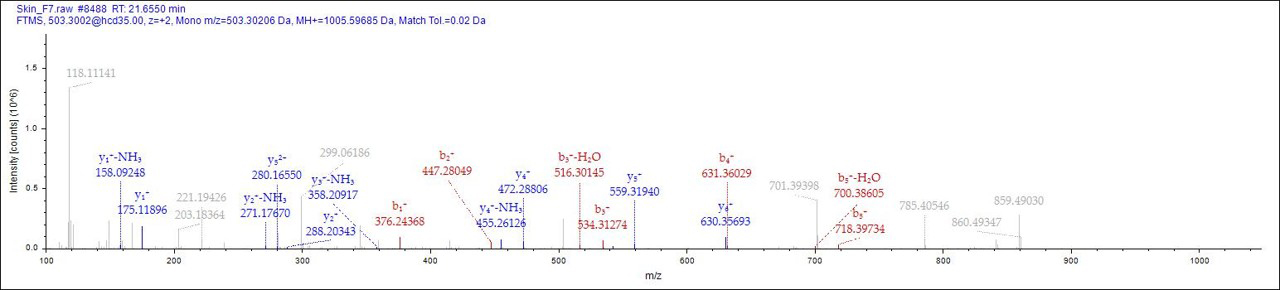


R.AASPSLR.H at m/z 503.30206

**Supplementary FIGURE S2.** Raw HPLC chromatograms of the clove extract (A) and the eugenol standard (B).

(A)

(B)
